# Coenzyme Q_0_ immobilized on Magnetic Nanoparticle: Synthesis and Antitumoral Effect on Saos, MCF7 and Hela Cell Lines

**DOI:** 10.22037/ijpr.2020.112680.13890

**Published:** 2020

**Authors:** Mohsen Aboukazempour Amiri, Mahmoud Reza Aghamaali, Hadi Parsian, Hamed Tashakkorian

**Affiliations:** a *Department of Biology, Faculty of Sciences, University of Guilan, Rasht, Iran. *; b *Department of Basic Sciences, Mazandaran University of Science & Technology (MUST), Babol, Iran. *; c *Cellular and Molecular Biology Research Center (CMBRC), Health Research Institute, Babol University of Medical Sciences, Babol, Iran. *; d *Department of Pharmacology, School of Medicine, Babol University of Medical Sciences, Babol, Iran.*

**Keywords:** Magnetic Nanoparticles, Coenzyme Q0, Cancer cells, AO/EB dual staining, Mtt assay

## Abstract

Many attempts in medical community focused on the preparation of anticancer agents. Various Coenzyme Q such as CoQ_0_ analogs have been reported as anti-inﬂammatory, anticancer, and antioxidant substances. In this study a novel derivatives of Coenzyme Q as an anticancer agent have been introduced. The prepared magnetic nanoparticle, containing CoQ_0_ were prepared using common chemical methods and also characterized by means of nuclear magnetic resonance (NMR), fourier transform infrared (FT-IR), thermal gravimetric analysis (TGA), and differential scanning calorimetric (DSC). To evaluate the antiproliferative effects of the nanoparticle, the prepared compound was treated with cell lines such as Hela, MCF-7 and Saos. Moreover, the outcomes were compared with normal fibroblast cell line. These assessments were performed by means of MTT assay. Investigation on the capability of this prepared nanoparticle showed some reliable results including cytotoxicities against MCF7, Saos and Hela cancer cell lines which were illustrated by displaying the morphology of the treated cells using AO/EB dual staining fluorescent technique. Employing simple method for preparation as well as the promising cytotoxic results makes it as a promising candidate for further bioexperiments.

## Introduction

In recent years cancer is one of the most common causes of death. The World Health Organization (WHO) statistics indicate that cancer mortality is increasing worldwide, with an estimation of 13 million deaths in 2030. Early and specific detection as well as systematic screening play a significant role in cancer prevention and treatment (1, 2). Primary treatments are included chemotherapeutic compounds and ionizing radiation for elimination of the cancer tumor mass. These treatments do not greatly differentiate between cancerous and normal cells, leading to systemic toxicity and adverse effects, limiting the maximum dose of drug (3). So, emerging novel therapeutic agents are highly required. Potential anticancer drugs need to be identified from alternative sources, such as natural products (4) and Peptide-based drugs (5, 6). Due to advantages of the peptide molecules, including small molecular size, high activity, low immunogenicity, diversity of sequence, appreciable biocompatibility and modification sites for possible functionalization, Peptide-based drugs gained much interest (7). Over the last two decades, a large number of nanoparticular drug delivery systems consisting of organic or inorganic materials have been evaluated for cancer therapy (8). The use of nanoparticles in drug delivery is a process of drug transition with an ultimate goal to deliver most of administered drug to the target sites, such as tumor tissues, while eliminating the drug accumulation at non-target sites. Thus, tremendous efforts have been made to develop targeted drug delivery systems during the past decade (9, 10). Nanocarrier (NC) drug delivery, such as liposome, polymeric nanoparticle and micelles, nanoshells, dendrimer, inorganic/metallic nanoparticles, and magnetic and bacterial nanoparticles, represents an exciting and promising strategy to effectively treat a wide range of diseases including cancer (11-13). 

Nanoparticles have many advantages as drug carrier agents; however, there are still several limitations, such as instability in circulation, distribution inadequate tissue, poor oral bioavailability, and toxicity (11). For tumor cell targeting, NCs have to conquer a series of obstacles, which can be classified as systemic targeting and intracellular targeting stages, to guide therapeutic agent to its therapeutic target (12). The systemic barriers include stability and survival in the bloodstream, prolonged circulation time, preferential surrounding and accumulation at tumor tissue, while the intracellular barriers involve the specific binding to the tumor cells and internalization of the drug-carriers by endocytosis, endo/lysosomal escape, cytosolic localization, and controllable drug release (14, 15). Based on their chemical composition, nanoparticles are broadly divided into two main groups: organic materials such as liposomes, carbon nanotubes, emulsions, dendrimers; and inorganic materials such as metals (16). 

Among the successes, magnetic nanoparticles (*e.g*., magnetite Fe_3_O_4_ or maghemite Fe_2_O_3_) appear to be quite suitable for being a drug targeted carrier, due to their capability of delivering pharmaceuticals to a specific site of the body by means of a gradient magnetic field (17-19). Iron oxide (or magnetite) nanoparticles with nanocrystalline magnetite (Fe_3_O_4_) cores due to their biocompatibility, facile synthesis, and biodegradability may be used for specific application and oncological medicine (20). The ability to quickly turn off drug delivery, by simple removing the external magnetic field is the most important advantage of magnetic drug targeting over conventional controlled release formulations (21). Also, magnetic nanoparticles can improve local action of the drug and minimize the side effects to the patient (22). Furthermore, a lot of unique properties, such as small size (~100 nm) allow them to function at the cellular level, super paramagnetism, high magnetization, and large specific surface area (23). 

Coenzyme Q (CoQ) is a well-known biomolecule, comprises of a quinone nucleus and a hydrophobic side chain containing variable number of trans isoprenoid units (24). Coenzyme Q_0_ (CoQ_0_, 2, 3-dimethoxy-5-methyl-1,4-benzoquinone), a novel quinone derivative without isoprenoid side chain, inhibits the activity of complex 1 of mitochondrial respiratory chain and prevent opening of the mitochondrial permeability transition pore (PTP) (25). Some studies reported that CoQ_0_ have a potent anti-oxidant effects and anti-cancer activity against human breast cancer cells through induction of apoptosis and cell-cycle arrest. The other researches presented potent cytotoxicity of CoQ_0_ towards insulin producing cells and also human breast cancer cells (26, 27). In spite of its cytotoxicity, some *in-vivo* studies have proved no injurious effects of CoQ_0_ when joined with other nutrients. Significantly, administration of CoQ_0_ mixture prevents oxidative damage in heart, blood, kidney, liver, and spleen of rodents (28, 29). The aim of this study was appraisal of the cytotoxic effects of synthesized nanoparticles containing Coenzyme Q_0_ on several cancer cell lines which include Fibroblast, Saos, MCF-7, and Hela cell lines.

## Experimental

Chemicals involving solvents, Coenzyme Q_0_ (2,3-Dimethoxy-5-methyl-p-benzo- quinone), ammonia solution (25%), (3-Mercaptopropyl) and trimethoxysilane were acquired from sigma (Switzerland) Company. Iron (II) chloride and Iron (III) chloride and tetraethyl ortho silicate were purchased from Merck (Germany) Company. All the chemicals were used without further purification.

*Equipment*

^1^HNMR spectrum was recorded on a Bruker 400 MHz. IR spectra were recorded on a Perkin-Elmer FT-IR-1710 spectrophotometer with the samples in KBr pellets. The plates of biological samples were read using Elisa reader (Ratio-China) at 570 nm. Biological results were calculated statistically using SPSS 17 and Microsoft Excel and presented as mean ± SD in triplicate experiment. Differences in the results were determined at significant difference of 0.05 (*P* *≤ 0.05*). FESEM was recorded on a Hitachi S4160 instrument (Tokyo, Japan). TGA were determined using Perkin-Elmer Pyris Diamond and Pyris 6 TGA Consumables, respectively under a nitrogen atmosphere at a heating rate of 10 ˚C min^-1^.

*Synthesis of conjugated 3-(trimethoxysilyl) propane-1-thiol to 2,* *3,5-trimethoxycyclohexa-2,* *5-diene-1,4-dione (Q*_0_*) (Silanized Q*_0_*):*

For the synthesis of the silanized Q_0_, firstly, 0.024 g Na was dissolved in 10 cc methanol and then 3-(trimethoxysilyl)propane-1-thiol (0.2 cc, 1.06 mmol) was added to the methoxide/methanol solution, and after 30 min Q_0_ (0.194 gr, 1.06 mmol) was added to the prepared mixture. Color of the solution was changed from light to dark brown. The reaction stayed at the same condition overnight under N_2_ atmosphere. Then, methanol was evaporated under reduced pressure and crude brown solid was washed at first with methanol to remove sodium methoxide and then with acetone for removal of excess raw materials to reach the pure silanized Q_0_ (0.3 gr).

IR (as KBr pellet): 683 cm^-1^ (C-S bond), 1112 cm^-1^ (broad peak related to C-O and Si-O bonds), 1250 cm^-1^ (C-C bond), 1331 cm^-1^ (CH_3_ groups), 1460 cm^-1^ (CH_2_ group), 1645 cm^-1^ (C = C bond), 1693 and 1750 cm^-1^ (C = O bond), 2930 cm^-1^ (C-H).

^1^HNMR: 0.91 ppm (t, CH_2_), 1.05 ppm (Q, CH_2_), 1.24 ppm (t, CH_2_), 1.65 ppm (s, Si-OMe), 3.17 ppm (s, -OMe), 3.56 ppm (s, -OMe), 3.75 ppm (d, C-H), 3.95 ppm (d, C-H).

*Preparation of Fe*_3_ *O*_4_ *-SiO*_2_ *(Silicated Fe*_3_ *O*_4_ *)*

For preparation of the silicated nanomagnetic particles, 2 gr Fe_3_O_4_ (22) was dispersed on 50 cc distilled water and sonicated for 15 min. Then, 1 cc TEOS solved in 9 cc distilled water, added to the aqueous mixture of the Fe_3_O_4_. 3 gr glycerol and also some drops of glacial acetic acid were added to the mixture to reach the pH 4.5. To reach the temperature up to 90 ºC, agitation of the mixture was continued for 2 h. Then, silicated nanomagnetic particles were removed by magnet and washed three times with water and acetone respectively to remove any non-supported material and reagents. The nanoparticles were dried in vacuum at 50 ºC overnight (2.8 gr). 

IR (as KBr pellet): 550-600 cm^-1^ (Fe-O-Fe bond), 1050-1100 cm^-1^ (broad peak related to Si-O bonds), 3400-3500 cm^-1^ (O-H bonds)

*Preparation of the immobilized Q*_0_* onto the silicated Fe*_3_*O*_4 _*(Fe*_3_*O*_4_*-SiO*_2-_*Q*_0_*) *

0.6 g of the silicated Fe_3_O_4_ was added to the 25 cc H_2_O and sonicated for 15 min. Then 0.3 gr (0.79 mmol) silanized Q_0_ that had been solved in 2 cc DMSO was added to the mixture. 1.5 cc glycerol and some drops of acetic acid were added to the above mixture to reach to the pH 4.5. After that, the mixture stirred for 2 h at 90 ºC. Then, by using magnet, the synthesized particles were collected and washed with DMSO, water, and acetone. At the end, wet powder was dried in vacuum oven at 40 ºC overnight (0.8 gr).

IR (as KBr pellet): about 630 cm^-1^ (Fe-O-Fe bond), 1038 cm^-1^ (broad peak related to C-O and Si-O bonds), 1425 cm^-1^ (CH_2_ group), 1632 cm^-1^ (C = C bond), 1745 cm^-1^ (C = O bond), 2925 cm^-1^ (C-H bond), 3421 cm^-1^ (O-H bond).

*Cell culture*

 The cell culture and primary human Fibroblasts isolation were performed according to the previous published article (28). Briefly, The HeLa (The human cervix cancer cell line), Saos (Sarcoma osteogenic cells), and MCF-7 (human breast adenocarcinoma cell line) were obtained from national cell bank of Iran (NCBI, Pasteur Institute of Iran). In 5% CO_2_ at 37 °C with 95% humidity of air, the cell lines were cultured in RPMI-1640 (Sigma-Aldrich, Germany) and fortified with 10% fetal bovine serum FBS (Gibco/Invitrogen), and 1% penicillin and streptomycin (Sigma-Aldrich, USA). After washing with PBS, the culture medium was changed each two days. This process was completed with 10 mL of fresh culture medium for 2-5 passage.

*MTT Assay*

The HeLa, MCF-7, and Saos cell lines were cultured in 96-well plates (10^4^ cells/well). Afterwards, 200 μL culture medium containing 10% (μg/mL) FBS was added to each wells. Under standard protocol, the culture medium was removed and the cells were treated with fresh ones containing different concentration of nanoparticles. After being in an incubator for 24, 48, and 72 h, 200 μL of the culture medium was removed and 50 μL of MTT (5mg/mL) solution was added to the wells. After appropriate time, the previous MTT solution was replaced with 200 μL of MTT solvent (acidic isopropanol) for solubilization of the formazan crystals. Subsequently, the mean absorbance of the plate was measured using elisa reader at 570 nm.

## Results

**Scheme 1.**
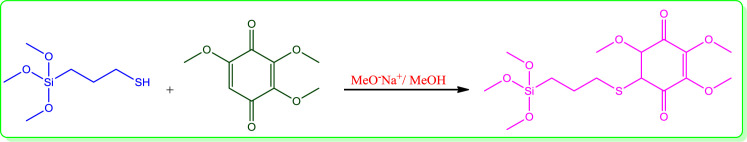
Synthesis of silanized Q_0_.

**Figure 1:**
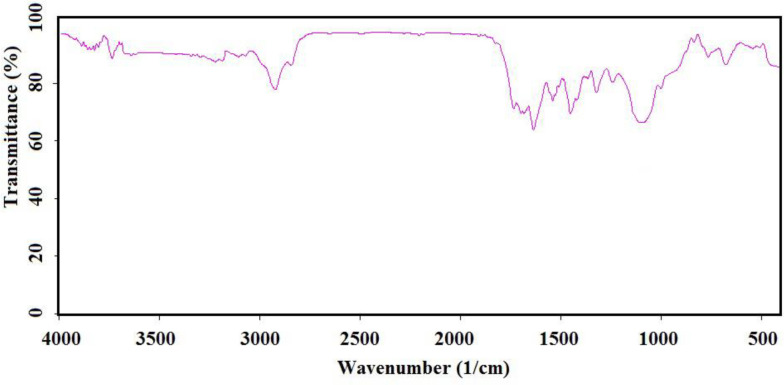
FTIR spectrum of the Silanized Q_0_

**Figure 2:**
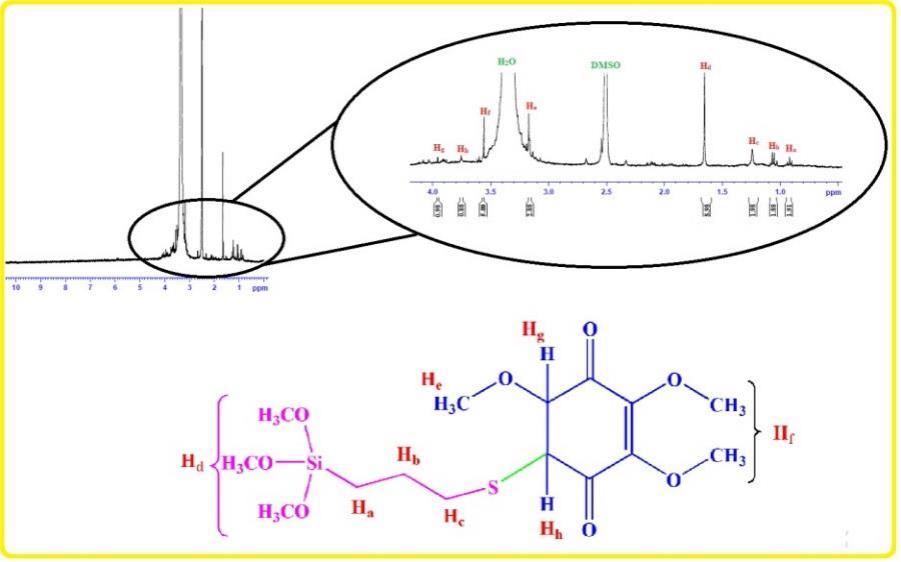
^1^HNMR of the synthesized Silanized Q_0_

Chemical preparation of the nanocomposite based material, specifically magnetic nanoparticles, is a known method for the introducing the novel material with fantastic properties. Moreover, immobilization of the biological materials onto the inorganic safe nanoparticles is a modern technique to save dosage in the ordinary usage for any targets. In this regard, the present project was studied for the preparation of the biologically significant compound, Q_0_, immobilized on the silanized Fe_3_O_4_ and studying of its corresponding anticancer activity. At the first, Q_0_ silanized was prepared by Michael addition of the 3-(trimethoxysilyl) propane-1-thiol to Q_0_ in the presence of sodium methoxide whose result was confirmed by FT-IR and NMR (Scheme 1). Its FT-IR spectrum (Figure 1) showed indexed peaks at 683 cm^-1^ (C-S bond), 1112 cm^-1^ (broad peak related to C-O and Si-O bond), 1250 cm^-1^ (C-C bond), 1331 cm^-1^ (CH_3_ groups), 1460 cm^-1^ (CH_2_ group), 1645 cm^-1^ (C = C bond), 1693 (C = O bond), 2930 cm^-1^ (C-H) and also, its NMR spectrum (Figure 2) with some peaks at 0.91 ppm (t, CH_2_), 1.05 ppm (Q, CH_2_), 1.24 ppm (t, CH_2_), 1.65 ppm (s, Si-OMe), 3.17 ppm (s, -OMe), 3.56 ppm (s, -OMe), 3.75 ppm (d, C-H), and 3.95 ppm (d, C-H) confirmed successful preparation of the Q_0_ silanized.

**Scheme 2:**
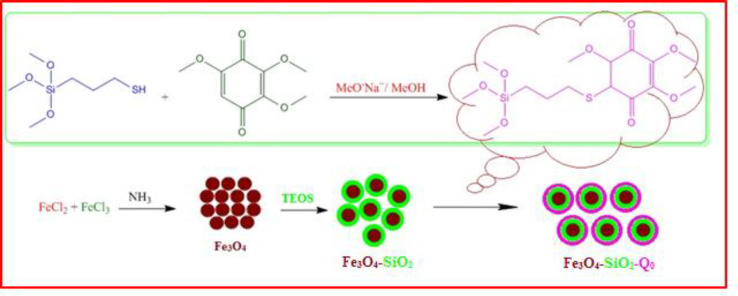
Synthesis of Fe_3_O_4_-SiO_2-_Q_0_

**Figure 3:**
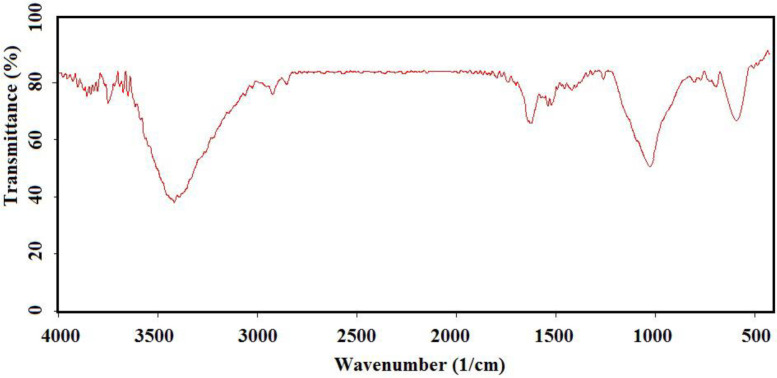
FTIR spectrum of the silicated Fe_3_O_4_

**Figure 4:**
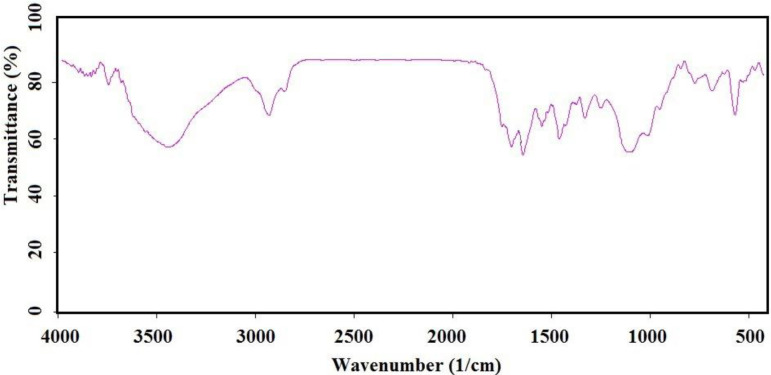
FTIR spectrum of the prepared magnetic nanoparticle functionalized with CoQ_0_

To have more functionalized nanoparticles, we coated the Fe_3_O_4_ nanoparticles with tetraethyl ortho silicate and then silanized CoQ_0_ was added. The FTIR spectrum of the coated magnetic nanoparticles was shown in (Figure 3). For preforming the last step, Q_0_ silanized was added to the prepared Fe_3_O_4_-SiO_2_ for chemical bonding and synthesis of the Fe_3_O_4_-SiO_2_-Q_0_ (Scheme 2). FT-IR of the synthesized compound (Figure 4) displayed characteristic peaks at about 630 cm^-1^ (Fe-O-Fe bond), 1038 cm^-1^ ( broad peak related to C-O and Si-O bonds), 1425 cm^-1^ (CH_2_ group), 1632 cm^-1^ (C = C bond), 1745 cm^-1^ (C = O bond), 2925 cm^-1^ (C-H bond), and 3421 cm^-1^ (O-H bond), that their correlations were reasonable and structure confirming.

*SEM and EDS*

**Figure 5:**
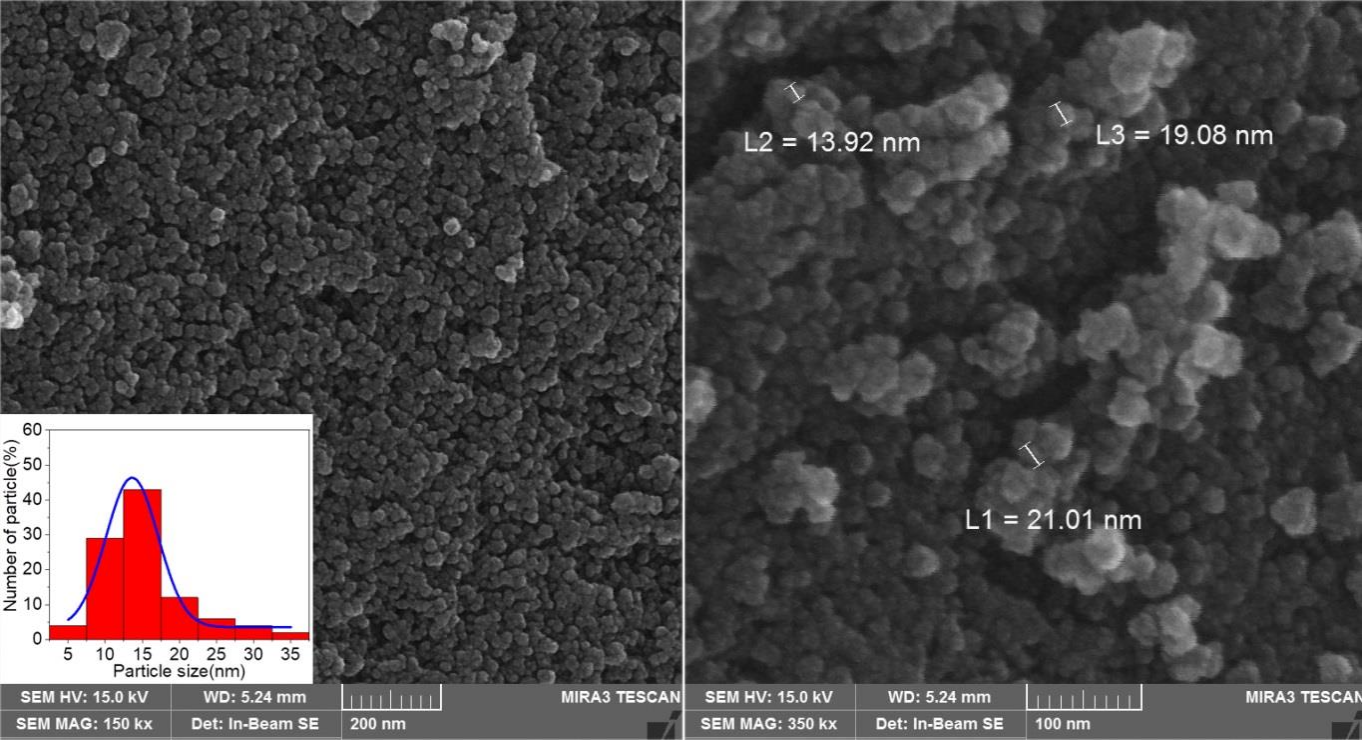
SEM of the magnetic nanoparticle in the scale of 200 and 100 nm

**Figure 6:**
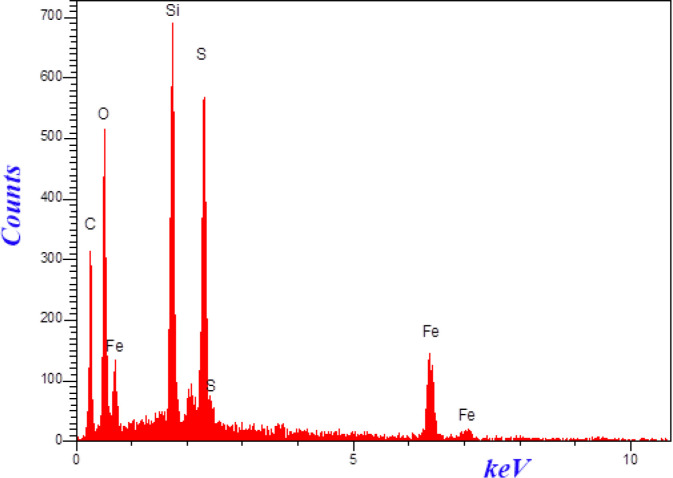
EDS of the nanoparticles containing CoQ_0_

The morphologies and size of synthesized materials Fe_3_O_4_-SiO_2-_Q_0_ was investigated by SEM. (Figure 5) demonstrated SEM images of Fe_3_O_4_-SiO_2-_Q_0 _nanocomposite in 200 and 100 nm magnification. It can be observed that the synthesized nanocomposite has a sphere like shape with diameter ranging below 50 nm which is significant for bioapplications. Also, EDS experiment which was shown in (Figure 6), clearly confirmed the presence of the mentioned elements in the final composite.

*Thermal properties*

*TGA and DTG*

**Figure 7:**
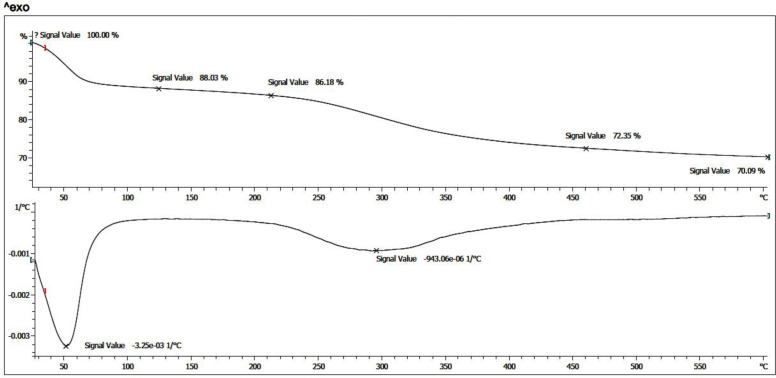
TGA and DTG curves of the synthesized magnetic nanoparticle

Thermogravimetric Analysis (TGA) of the prepared nanoparticle was evaluated. After salinization of the magnetic nanoparticles and further immobilization of CoQ_0_, thermal analysis was performed under N_2_ atmosphere, from room temperature to 600 °C. As presented in (Figure 7), also, the Derivative Thermogravimetric Analysis (DTG) curve for both thermogram exhibited that the prepared nanoparticle has one main stage weight loss more than solvent removal stage below 100 °C at around 280 ˚C. This weight loss is due to the presence of organic structures in the nanoparticles which were around 27.5%. Moreover, according to the TGA curves, the amount of inorganic excess materials containing Fe_3_O_4_ and silane groups was around 72.5%. 

The effects of nanoparticles containing CoQ_0_ on the proliferation of cancer cell lines (Fibroblast, Saos, MCF-7 and Hela) were investigated by MTT assay.

*Inhibition of cell proliferation by CoQ*_0_ *on fibroblast cells*

**Figure 8:**
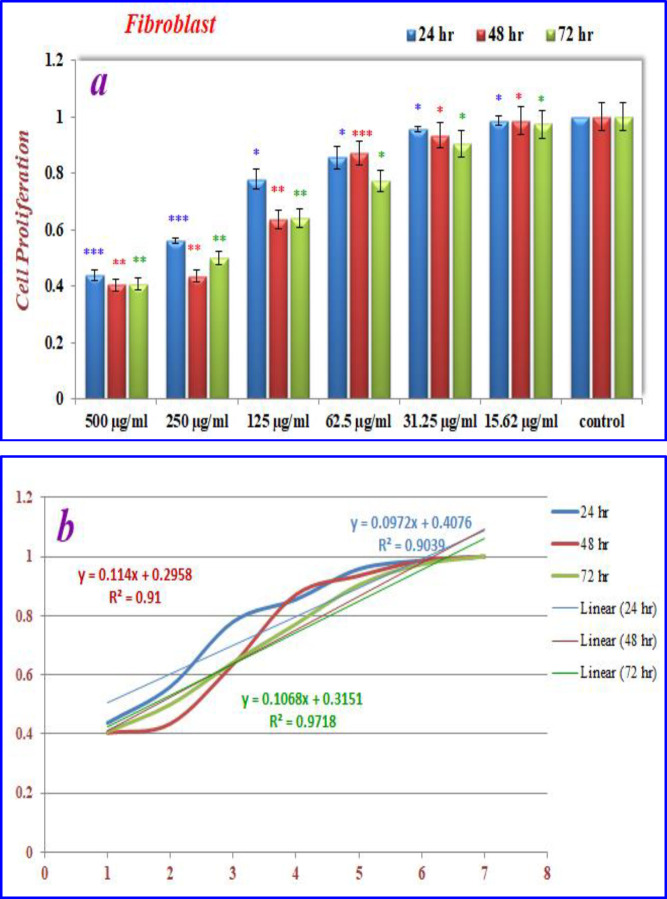
(a) The inhibition of cell proliferation by different concentrations of nanoparticles containing CoQ_0_ as compared to the control cells in 24, 48 and 72 h. Each bar represents the mean ± standard deviation of three independent tests. *P < .05; **P < .01; ***P < 001 compared with untreated control cells.

**Figure 9:**
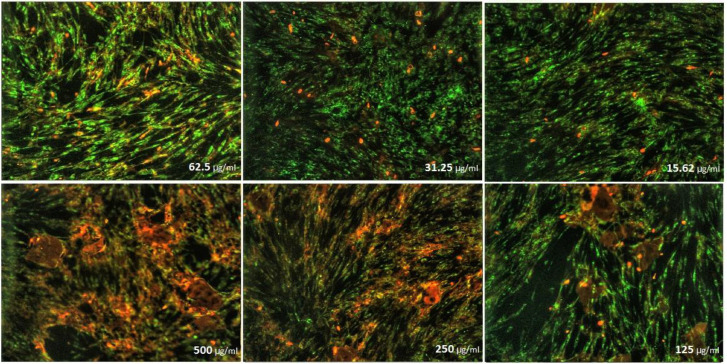
Cell Morphology: Evaluation of apoptosis incidence and morphological observation of Fibroblast treated with nanoparticles containing CoQ_0_ using AO/EB staining at × 400 magnifications. Early apoptotic cells (chromatin condensation stained green); late apoptotic cells (chromatin condensation stained orange); membrane blebbing; shrunken and loss of membrane shapes are can be seen in the (Figures). The higher the concentration of CoQ_0_ nanoparticles used the more aggressive induction of death in the cancer cells

As can be seen in (Figures 8), cell proliferation of the treated fibroblast cells with nanoparticles containing CoQ_0_ was decreased. This effect resulted in the morphological changes (Figure 9) after 24, and 72 h in a dose dependent manner. Fibroblast cells that exposed to nanoparticles containing CoQ_0_ exhibited an IC_50_ about 250 µM for all three mentioned times as compared to the control cells (Nanoparticles without CoQ_0_).

**Figure 10:**
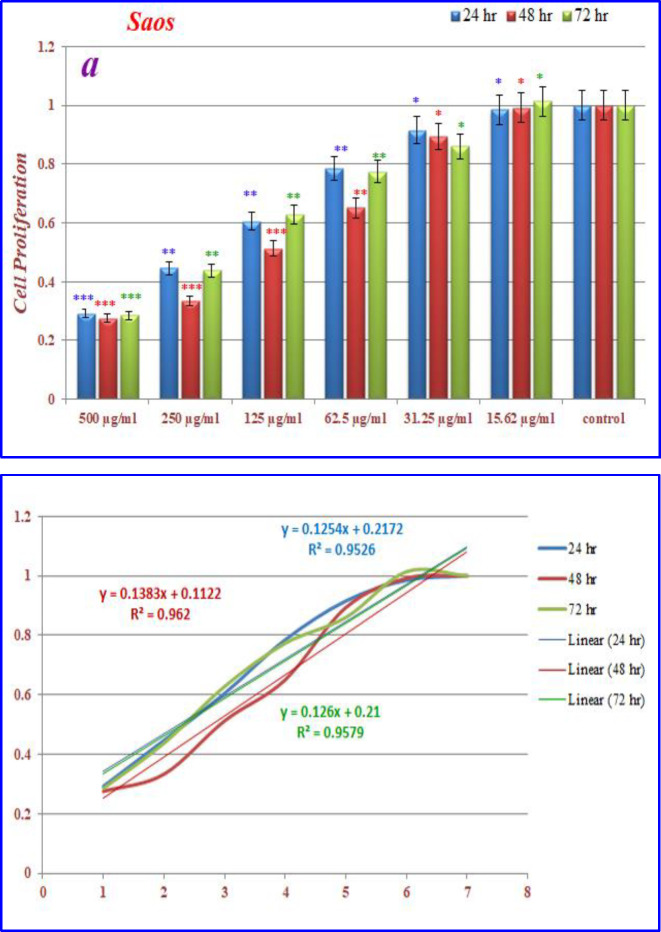
The inhibition of cell proliferation by different concentrations of nanoparticles containing CoQ_0_ as compared to the control cells in 24, 48 and 72 h. Each bar represents the mean ± standard deviation of three independent. *P < 05; **P < 01; ***P < 001 compared with untreated control cells

**Figure 11:**
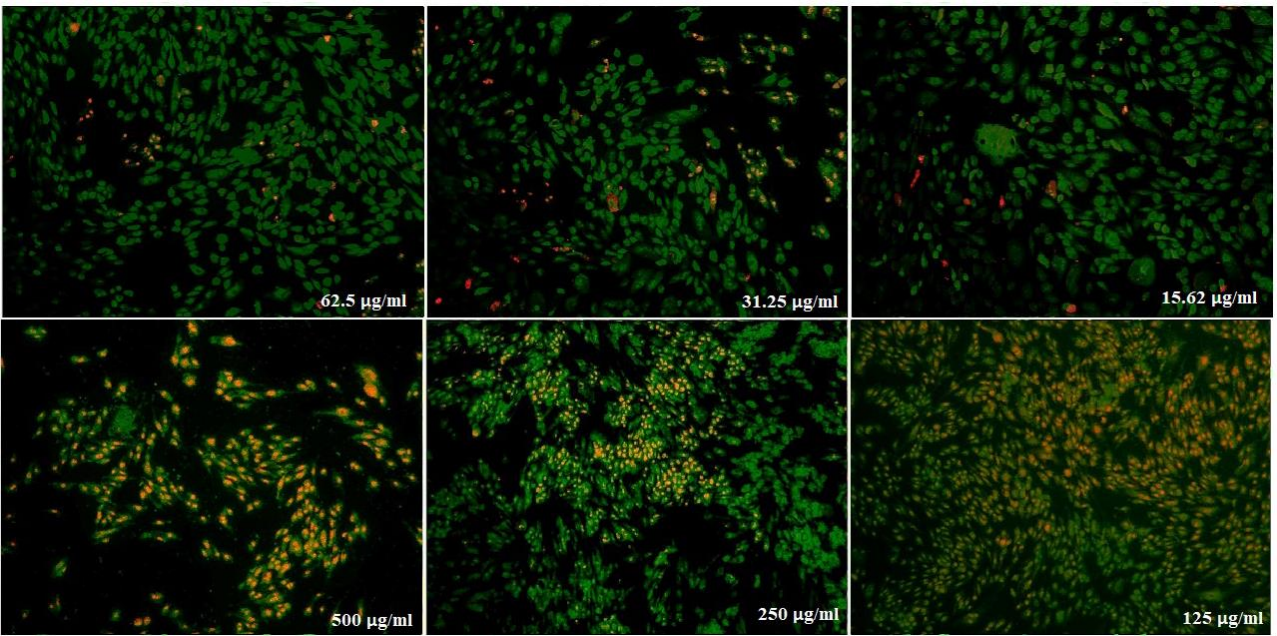
Cell Morphology:  Evaluation of apoptosis incidence and morphological observation of Saos treated with nanoparticles containing CoQ_0_ using AO/EB staining at × 400 magnifications. Early apoptotic cells (chromatin condensation stained green); late apoptotic cells (chromatin condensation stained orange); membrane blebbing; shrunken and loss of membrane shapes are can be seen in the Figures. The higher the concentration of CoQ_0_ nanoparticles used the more aggressive induction of death in the cancer cells

*Inhibition of cell proliferation by CoQ*_0_ *on saos cells*

(Figure 10) displays the treated saos cells with nanoparticles, containing CoQ_0_ which resulted in the significant decrease in cell proliferation. This reduction resulted in morphological changes after 24, 48, and 72 h in a dose dependent manner. Saos cells, exposed to nanoparticles containing CoQ_0_ exhibiting an IC_50_ about 250 µM for 24 and 72 h and an IC_50_ about 125 µM for 48 h as compared to the control cells. Evaluation of apoptosis incidence and morphological observation of Saos cells treated with nanoparticles was conducted using AO/EB staining technique (Figure 11).

**Figure 12:**
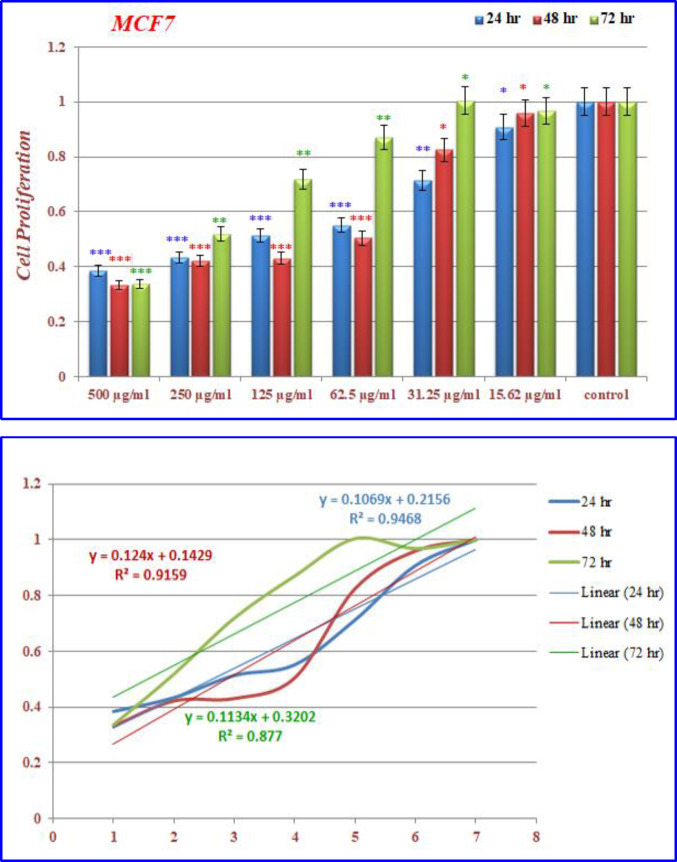
The inhibition of cell proliferation by different concentrations of nanoparticles containing CoQ_0_ as compared to the control cells in 24, 48 and 72 h. Each bar represents the mean ± standard deviation of three independent. *P < 05; **P < 01; ***P < 001 compared with untreated control cells

**Figure 13:**
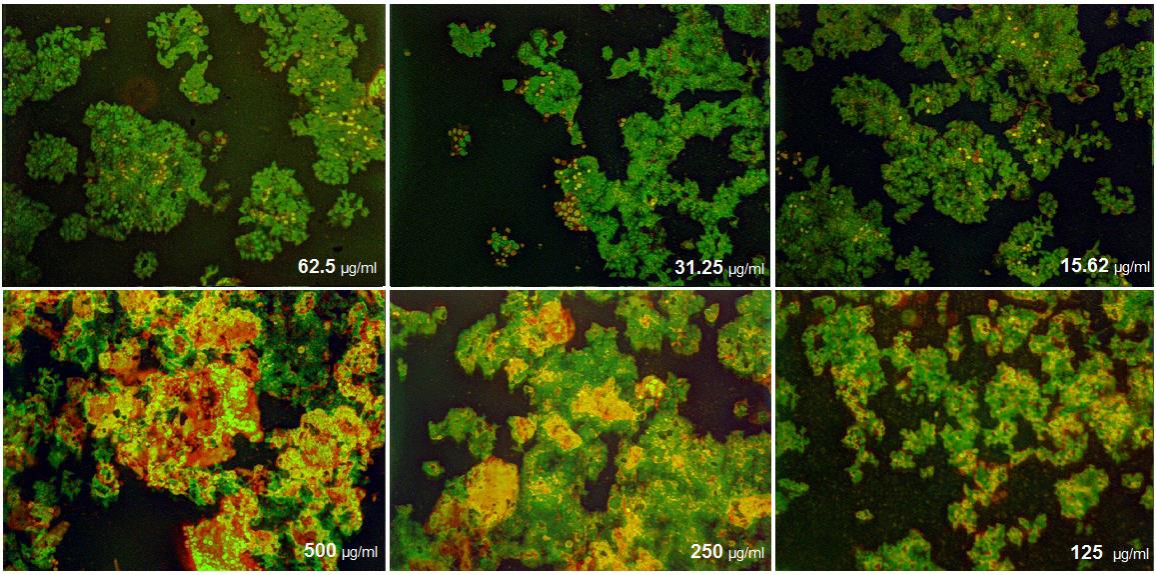
Cell Morphology: Evaluation of apoptosis incidence and morphological observation of MCF7 treated with nanoparticles containing CoQ_0_ using AO/EB staining at × 400 magnifications. Early apoptotic cells (chromatin condensation stained green); late apoptotic cells (chromatin condensation stained orange); membrane blebbing; shrunken and loss of membrane shapes are can be seen in the Figures. The higher the concentration of CoQ_0_ nanoparticles used the more aggressive induction of death in the cancer cells

*Inhibition of cell proliferation by CoQ*_0_ *on MCF-7 cells*

(Figures 12) demonstrated that the treated MCF-7 cells with nanoparticles containing CoQ_0_ caused the reduction in cell proliferation. This dose dependent effect resulted in morphological changes and evaluated using AO/EB staining technique (Figure 13). MCF-7 cells, exposed to nanoparticles containing CoQ_0_ exhibited IC_50_ about 62.5 µM for 24 and 48 h and an IC_50_ about 250 µM for 48 h as compared to the control cells.

**Figure 14:**
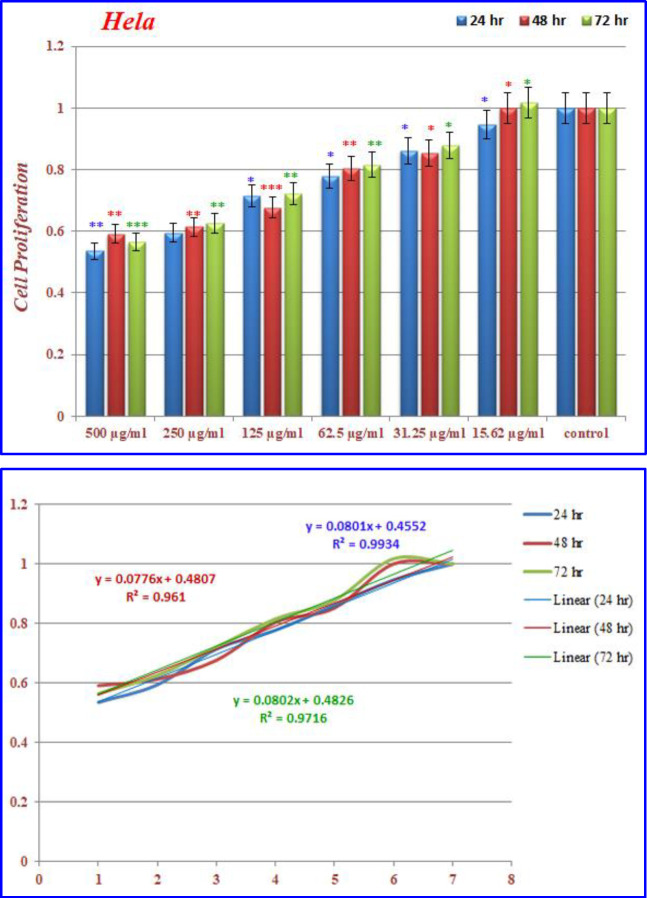
The inhibition of cell proliferation by different concentrations of nanoparticles containing CoQ0 as compared to the control cells in 24, 48 and 72 h. Each bar represents the mean ± standard deviation of three independent. *P < 05; **P < 01; ***P < 001 compared with untreated control cells

**Figure 15:**
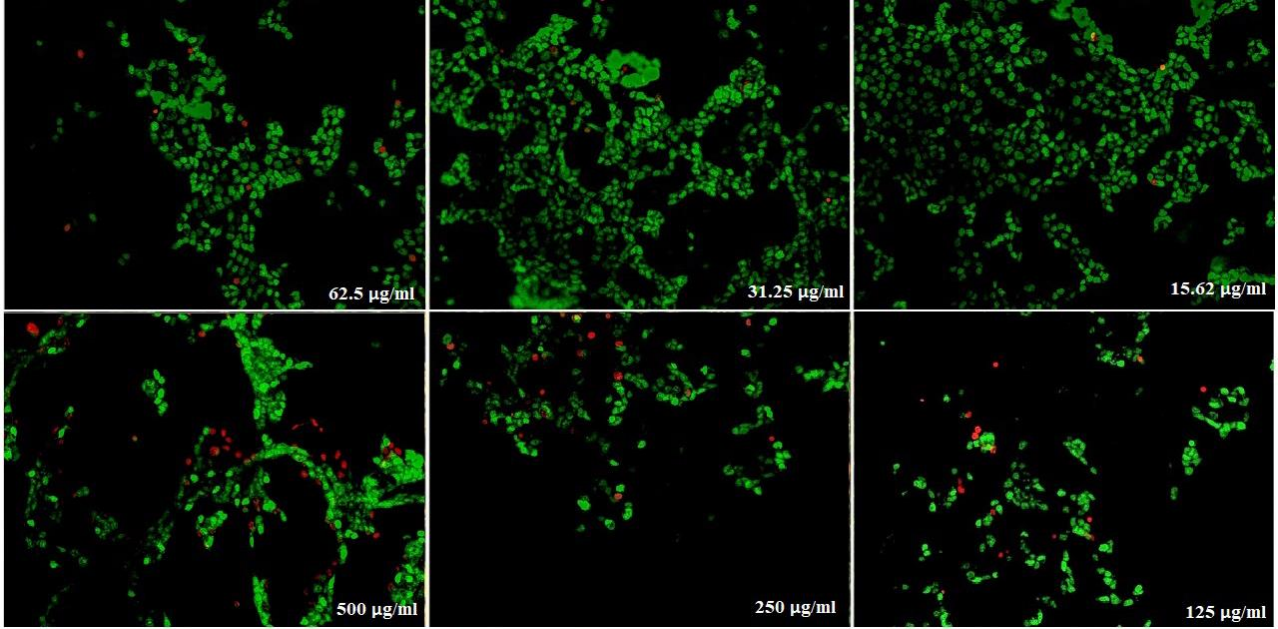
Cell Morphology: Evaluation of apoptosis incidence and morphological observation of Hela treated with nanoparticles containing CoQ_0_ using AO/EB staining at × 400 magnifications. Early apoptotic cells (chromatin condensation stained green); late apoptotic cells (chromatin condensation stained orange); membrane blebbing; shrunken and loss of membrane shapes are can be seen in the Figures. The higher the concentration of CoQ_0_ nanoparticles used the more aggressive induction of death in the cancer cells

*Inhibition of cell proliferation by CoQ*_0_ *on Hela cells*

)Figures 14( show the treated Hela cells with nanoparticles containing CoQ_0._ The decrease in cell proliferation consequently resulted in morphological changes after 24, 48, and 72 h in a dose dependent manner. Hela cells, exposed to nanoparticles containing CoQ_0_ exhibited an IC_50_ about 500 µM in all of the three mentioned times as compared to the control cells. Evaluation of apoptosis incidence and morphological observation of Hela cells treated with nanoparticles containing CoQ_0_ conducted using AO/EB staining technique (Figure 15).

## Discussion

Due to the fact that cancer is one of the most common causes of death in the world, many efforts in the medical community have focused on the development of anticancer drugs (30). Currently, the cancer is treated with surgery, radiotherapy, chemotherapy, targeted therapy, hormonal therapy, and immunotherapy modalities, but all of them carry some side effects and shortcomings such as drug resistance, severe adverse reactions, moderate therapeutic efficacy, low concentration of therapeutics at the site of action and/or fast renal clearance of conventional drugs, etc. The reason for these shortcomings and side-effects are mostly due to non-speciﬁcity and systemic toxicity of anti-cancer drugs(31, 32). 

Over the past few years, the use of nanoparticles has gained tremendous interest, because nanoparticles, by using both passive and active targeting strategies, can enhance the intracellular concentration of drugs in cancer cells while avoiding toxicity in normal cells (11, 33). Various coenzyme Q (CoQ) analogs have been reported as anti-inﬂammatory, anticancer, and antioxidant substances. Coenzyme Q_0_ (CoQ_0_ or Ubiquinone 0) is a redox-active ubiquinone compound that accumulates predominantly in mitochondria and also is a potent inhibitor than all other quinone analogs (34). In previous *in-vitro* and *in-vivo* studies have shown that CoQ_0_ has many biological effects on cancer cells, such as cell cycle arrest, apoptosis, and anti tumorigenic activity (26). 

**Figure 16:**
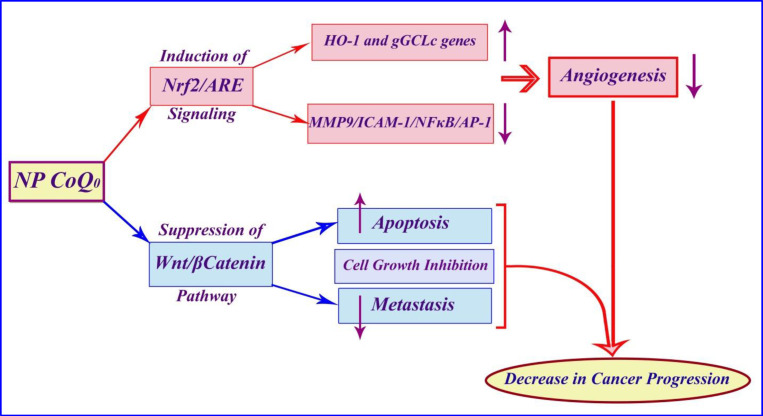
The proposed mechanism of anticancer activity of Coenzyme Q0 immobilized on magnetic nanoparticle against cancer cells

In this study, we investigate the anticancer effect of nanoparticles containing CoQ_0_ on some different common cancer cell lines. The results of the present study indicated that the nanoparticles containing CoQ_0_ could be an anti-cancer agent according to the proposed schematic mechanism of the anticancer activity of Coenzyme Q0 immobilized on magnetic nanoparticle (Figure 16).

Furthermore, the immobilized CoQ_0_ has shown a strong potential anti proliferative effects on several cancer cell lines as reported by the other investigators (26). Dose and time dependency as well as the effect of the types of the cell lines on cells proliferation through the MTT assay were investigated in this article.

CoQ_0_ treatment also resulted in the decrease in cell viabilities in A549 (lung cancer cells), HepG2 (HepG2 hepatoma cells), and SW480 (colon cancer cells) cancer cell lines. Moreover, study on A549 lung cancer cells indicated that CoQ_0_ induced reactive oxygen species (ROS) generation and apoptosis in A549 cells, inhibited by the antioxidant ascorbic acid (35). 

 Our findings demonstrated a reduction in cells viabilities, when compared with the control cells after treatment with different doses of nanoparticles in several cancer cell lines for 24, 48, and 72 h. The increase in concentrations of the nanoparticles containing CoQ_0_ samples will result in the significant decrease in proliferation of the all mentioned cancer cell lines. 

In addition, it is reported that cell viabilities dramatically decreased when the MCF-7 cells were treated with CoQ_0_. CoQ_0_ in the presence of ultraviolet B induced apoptosis in human breast cancer cells. The treatment using CoQ_0_ moderately inhibits the growth of breast cancer MCF-7 cells, and the cell viability was significantly decreased when the cells were pretreated with UVB irradiation (29). 

Several studies have examined various mechanisms for CoQ_0_ performance. A study showed that non-cytotoxic concentration of CoQ_0_ exhibits anti-inﬂammatory and antioxidant properties against LPS (lipopolysaccharide) challenge and pharmacological actions of CoQ_0_ are mediated via inhibition of NFκB/AP-1 activation and induction of Nrf2/AREsignaling. MTT result of another study showed that CoQ_0_ treatment for 24 h did not affect the viability of macrophages up to 10 μM concentration, whereas a signiﬁcant reduction (p b 0.05) was observed with 15 and 20 μM concentrations (27). 

Published studies showed that efficacy of CoQ_0_ in cell growth inhibition, induction of apoptosis, and prevention of metastasis may be due to suppression of the Wnt/β-catenin signaling pathway in melanoma cells. Additionally, according to the previous articles, effects of CoQ_0_ on the proliferation of murine melanoma cell lines (B16F10, B16F1, and A2058) were determined by MTT assay. CoQ_0_ exhibits a significant cytotoxic effect on melanoma cell lines (B16F10, B16F1, and A2058), while causing little toxicity toward normal (HaCaT) cells. These results demonstrate that CoQ_0_ were more potent towards the cancer cells than the normal ones.^(^^31^^)^ In addition, previous studies have indicated an important issue that CoQ_0_ through suppression of MMP-9/ICAM-1/NF-kB, activation and up regulation of HO-1 and g-GCLC genes, both via Nrf2/ARE signaling cascades in TNF-α-activated human endothelial cells, causes anti-angiogenic properties. Cell viability results from the MTT tested concentrations (2.5, 5 and 10 mM) in a study showed that up to 10 mM of CoQ_0_ had no adverse effects on endothelial cell number (28). 

It is reported that MCF-7 cells were exposed to 0 to 35 µM of CoQ_0_ for 24 to72 h. Treating MCF-7 cells with 35 µM CoQ_0_ for 24 and 48 h had no significant effects on cell viabilities. However, cell viability dramatically decreased when the MCF-7 cells were treated with CoQ_0_ (35 µM) for 72 h (29). 

## Conclusion

In recent years, researchers have focused on the preparation of derivatives of biomolecules. Incorporating nanoparticles as a biomolecule’s base, can exhibit some promising characteristics. On the basis of this hypothesis, we designed a study to survey on the cytotoxic characteristics of magnetic nanoparticles containing CoQ_0_. After preparation of the mentioned nanoparticles and appraisal of the structural characterization, by incorporation of the MTT assay, findings indicate that nanoparticles containing CoQ_0_ have cytotoxic effects on several known cancer cell lines when compared with the nanoparticles without CoQ_0_. Evaluation of apoptosis incidence and morphological observation were also detected by AO/EB staining method. Moreover; thermophysical properties were investigated thoroughly. These results demonstrated that these magnetric nanoparticles have noteworthy potential which makes it a trustworthy compound for certain bioapplications. 
